# Terpenoid natural products exert neuroprotection *via* the PI3K/Akt pathway

**DOI:** 10.3389/fphar.2022.1036506

**Published:** 2022-10-13

**Authors:** Bingyao Xu, Lan Bai, Lu Chen, Rongsheng Tong, Yibin Feng, Jianyou Shi

**Affiliations:** ^1^ Personalized Drug Therapy Key Laboratory of Sichuan Province, Department of Pharmacy, Sichuan Academy of Medical Science and Sichuan Provincial People’s Hospital, University of Electronic Science and Technology of China, Chengdu, China; ^2^ The State Key Laboratory of Southwestern Chinese Medicine Resources, Department of Pharmacy, Chengdu University of Traditional Chinese Medicine, Chengdu, China; ^3^ School of Chinese Medicine, Li Ka Shing Faculty of Medicine, The University of Hong Kong, Hong Kong SAR, China

**Keywords:** natural products, neuroprotection, PI3K/Akt pathway, derivatives, terpenoids

## Abstract

PI3K/Akt, an essential signaling pathway widely present in cells, has been shown to be relevant to neurological disorders. As an important class of natural products, terpenoids exist in large numbers and have diverse backbones, so they have a great chance to be identified as neuroprotective agents. In this review, we described and summarized recent research for a range of terpenoid natural products associated with the PI3K/Akt pathway by classifying their basic chemical structures of the terpenes, identified by electronic searches on PubMed, Web of Science for research, and Google Scholar websites. Only articles published in English were included. Our discussion here concerned 16 natural terpenoids and their mechanisms of action, the associated diseases, and the methods of experimentation used. We also reviewed the discovery of their chemical structures and their derivatives, and some compounds have been concluded for their structure–activity relationships (SAR). As a result, terpenoids are excellent candidates for research as natural neuroprotective agents, and our content will provide a stepping stone for further research into these natural products. It may be possible for more terpenoids to serve as neuroprotective agents in the future.

## Graphical Abstract

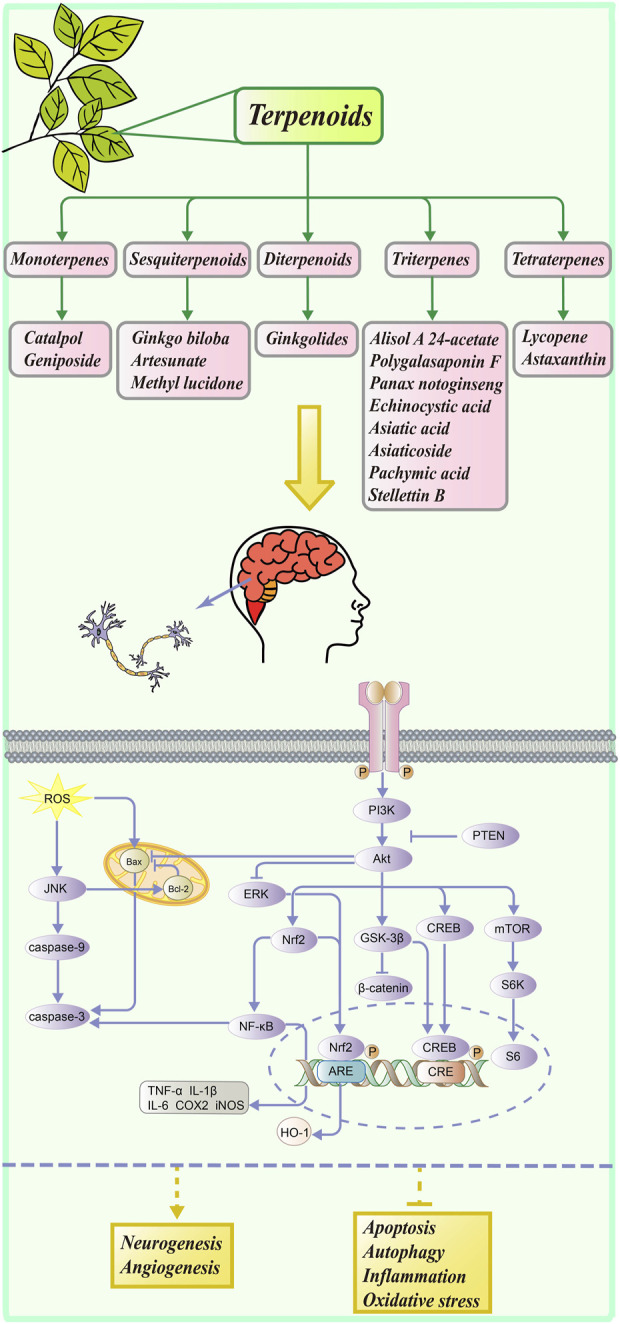

## Introduction

The brain is a complex and fragile system closely related to our studies and life. By altering its structure and function, the brain can store information, but it is also susceptible to damage, invasion by pathogens, and neurodegeneration (Heneka et al., 2018). Neurodegenerative disease (NDD) is caused by degeneration and subsequent loss of neurons because nerve cells cannot regenerate or replace. Gradually, the patient’s cognition and peripheral and central nervous system functions will decrease (Fakhri et al., 2021). It is widely recognized that neurodegenerative diseases include Alzheimer’s disease (AD), Parkinson’s disease (PD), Huntington’s disease (HD), and diseases affecting the central nervous system (CNS) (Gitler et al., 2017; Ahmed et al., 2018; Li et al., 2021). In recent years, with an aging population worldwide, neurological diseases have become a major health concern and economic burden, posing a severe threat to human health. Some researchers have even predicted that the number of patients with AD will exceed 100 million by the year 2050 (Prince et al., 2013; Pohl and Kong Thoo Lin, 2018). However, as of today, these nerve diseases cannot be radically cured, instead relying solely on applicable drugs to alleviate their symptoms.

In ancient times, plants were used as medicine, and natural products of all kinds were used as medicine, including plants, animals, microbes, and marine organisms (Shi et al., 2010b; Chen et al., 2021b). Natural products exhibit a high level of biological activity due to their unique chemical diversity, which is crucial to the development of new lead compounds and chemical molecular scaffolds. In the process of discovering new drugs, natural products have offered abundant chemical structures and new pharmacological mechanisms. With their significant curative properties and small side effects, natural products have become one of the major sources of drug discovery in our time. In the past few years, the FDA has approved more than 60% of natural medicines (Galm et al., 2007; Rodrigues et al., 2016; Yuan et al., 2016). Among these natural medicines is galantamine, which has been approved by the FDA as an anti-AD medicine isolated from the bulbs and flowers of snowdrop *Galanthus woronowii* (Amaryllidaceae) (Yoo and Park, 2012). A number of synthetic drugs can also be prescribed to treat nervous system issues, including idebenone, diazepam, doxepin, and memantine hydrochloride, but these drugs all have unwanted side effects. Thus, developing natural neuroprotective agents with high efficacy and low toxicity may increase the therapeutic prospects for neurological disorders (Chen et al., 2021c).

Terpenoids, among the largest natural product families, possess complex chemical structures and play a significant role in the drug discovery of NDD. Based on the number of units of the isoprene unit (C5), terpenes can be classified as hemi- (C5), mono- (C10), sesqui- (C15), di- (C20), sester- (C25), tri- (C30), tetra- (C40), or polyterpenes (*n* > 8). In addition, many related modification products and derivatives have also been characterized. Moreover, terpenoids have been reported to have a number of benefits, including potential anti-inflammatory, anti-carcinogenic, and neuroprotective effects, which may lead to improved human health (Kiyama, 2017).

**FIGURE 1:**
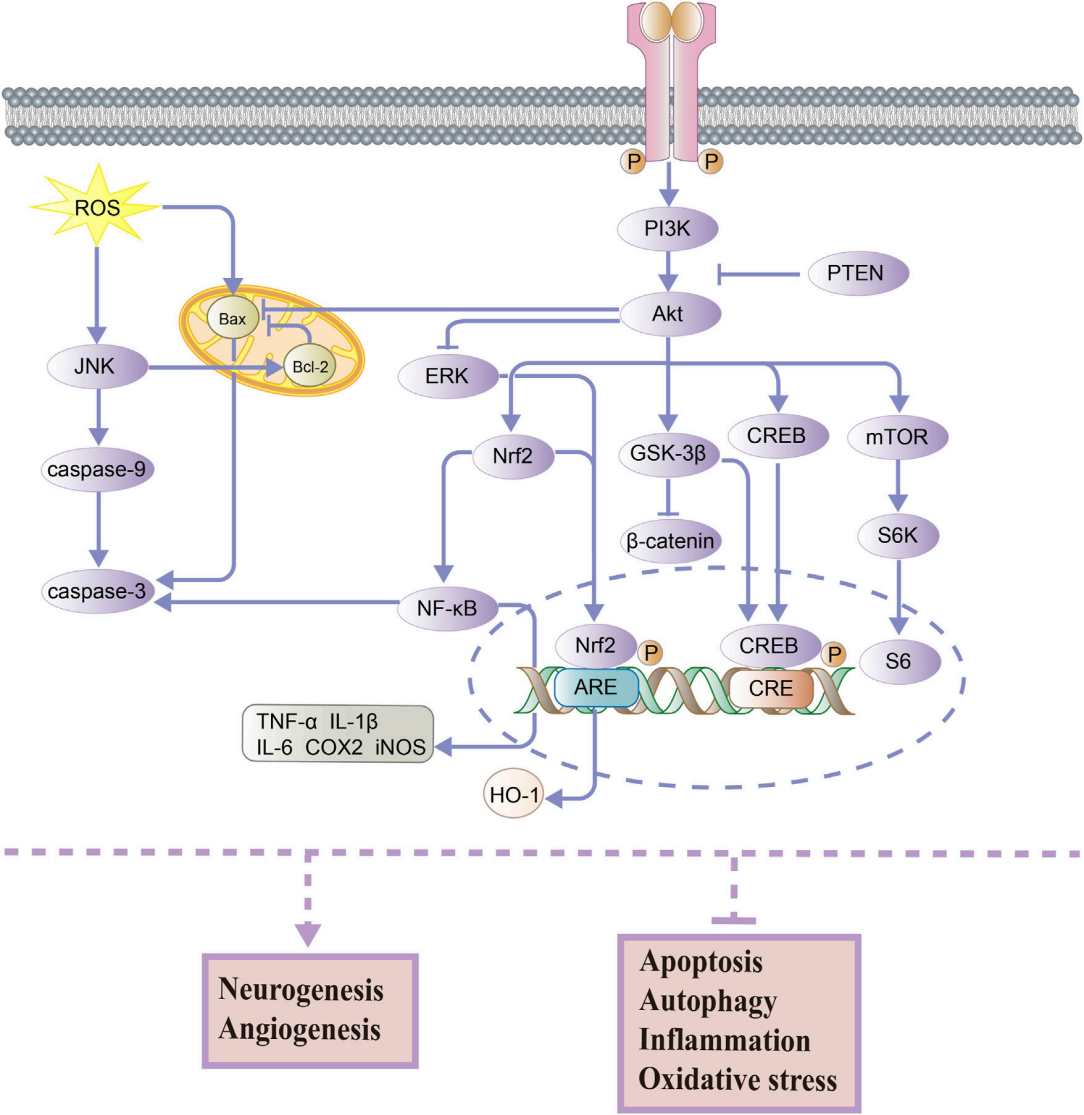
PI3K/Akt signaling pathway for neuroprotection.

In the nervous system, the PI3K/Akt pathway is an important signaling pathway that contributes to neuronal growth, proliferation, survival, and function at all stages of development (Coelho and Leevers, 2000; Huang et al., 2005). In the downstream of PI3K, Akt is a key target in mediating anti-cell apoptosis and regulating cellular metabolism (Manning and Cantley, 2007). As shown in Figure 1, PI3K can be activated and promote the phosphorylation of Akt, which regulates the phosphorylation of various downstream proteins and ultimately affects the activity of several cellular physiological processes. It should be noted that PTEN (phosphatase and tensin homolog deleted on chromosome 10) can downregulate the PI3K/Akt signaling pathway (Chen et al., 2021a). Then, the activation of Akt leads to the nuclear translocation of Nrf2 and p-CREB, which promotes cell survival and prevents apoptosis. The transcription factor Nrf2 (nuclear factor-erythroid 2-related factor 2) regulates the transcription of antioxidant genes such as HO-1 (heme oxygenase-1) and inhibits the oxidative stress response (Li et al., 2016). High activity of Nrf2 also has the ability to downregulate the NF-κB pathway, which is associated with anti-inflammatory and antioxidant activities. Also, CREB (cAMP-response element-binding protein) is a transcription factor that promotes the expression of neuroprotective proteins (Belgacem and Borodinsky, 2017). It is well known that the Wnt/β-catenin signaling pathway is vital in regulating brain development, growth, and differentiation (Kurimoto et al., 2015). Its downstream effector, GSK-3β (glycogen synthase kinase-3 beta), is activated by Akt, which inhibits the β-catenin expression and reduces neuronal activity. Moreover, AKT can promote mTOR (mammalian target of rapamycin) expression, which regulates cellular nutrition and energy supply and stimulates protein synthesis (Ling et al., 2019). Akt can also inhibit the expression of the pro-apoptotic protein Bax (BCL2-associated X) and increase cell survival, as well as increase the expression of the anti-apoptotic protein Bcl-2 (B-cell lymphoma 2) and reduce apoptosis in cells. In general, PI3K and Akt are known to activate several substrates and downstream effectors involved in metabolism, cell survival and apoptosis, differentiation, and proliferation (Hannan et al., 2020).

We classified terpenoids according to their chemical structure and searched numerous academic websites for articles on terpenoid natural products related to the neuroprotective pathway PI3K/Akt. We reviewed their neuroprotective activities *via* the PI3K/Akt pathway, some of these terpenoid derivatives, and their structure–activity relationship studies. Hopefully, our study will inspire researchers and promote the development of terpenoid neuroprotective agents.

## Monoterpenes

Monoterpenes are isoprene dimers with the composition C10H16. Catalpol and geniposide described in the following sections are both iridoids that belong to special monoterpenoids. Usually, they are linked with sugars to form glycosides, which are collectively referred to as iridoid glycosides.

### Catalpol and its derivatives

Catalpol is a monoterpene containing an aldehyde acetal, which belongs to the iridoids. Having been isolated from plants in 1888, it has been extensively studied for its anti-inflammatory, antioxidant, and other biological properties (Claassen, 1888; Villasenor and Chemistry, 2007). The primary active ingredient in Rehmanniae Radix is catalpol, which prevents multiple central nervous system diseases (Jiang et al., 2015). Catalpol has been found to provide antioxidant and neuroprotective effects on mouse models of depression, improving their depressive behavior by upregulating the PI3K/Akt/Nrf2/HO-1 signaling pathway, indicating that this pathway could be used as a potential target and biomarker for the treatment of depression with catalpol (Wang et al., 2021a; Wu et al., 2021). In addition, some studies have reported that catalpol activated the PI3K/Akt/mTOR pathway, decreasing the expression of miR-124 and increasing the expression of downstream protein S6, thus enhancing *in vivo* axon growth and neuronal survival in stroke models (Wang et al., 2019; Zhu et al., 2019).

**FIGURE 2:**
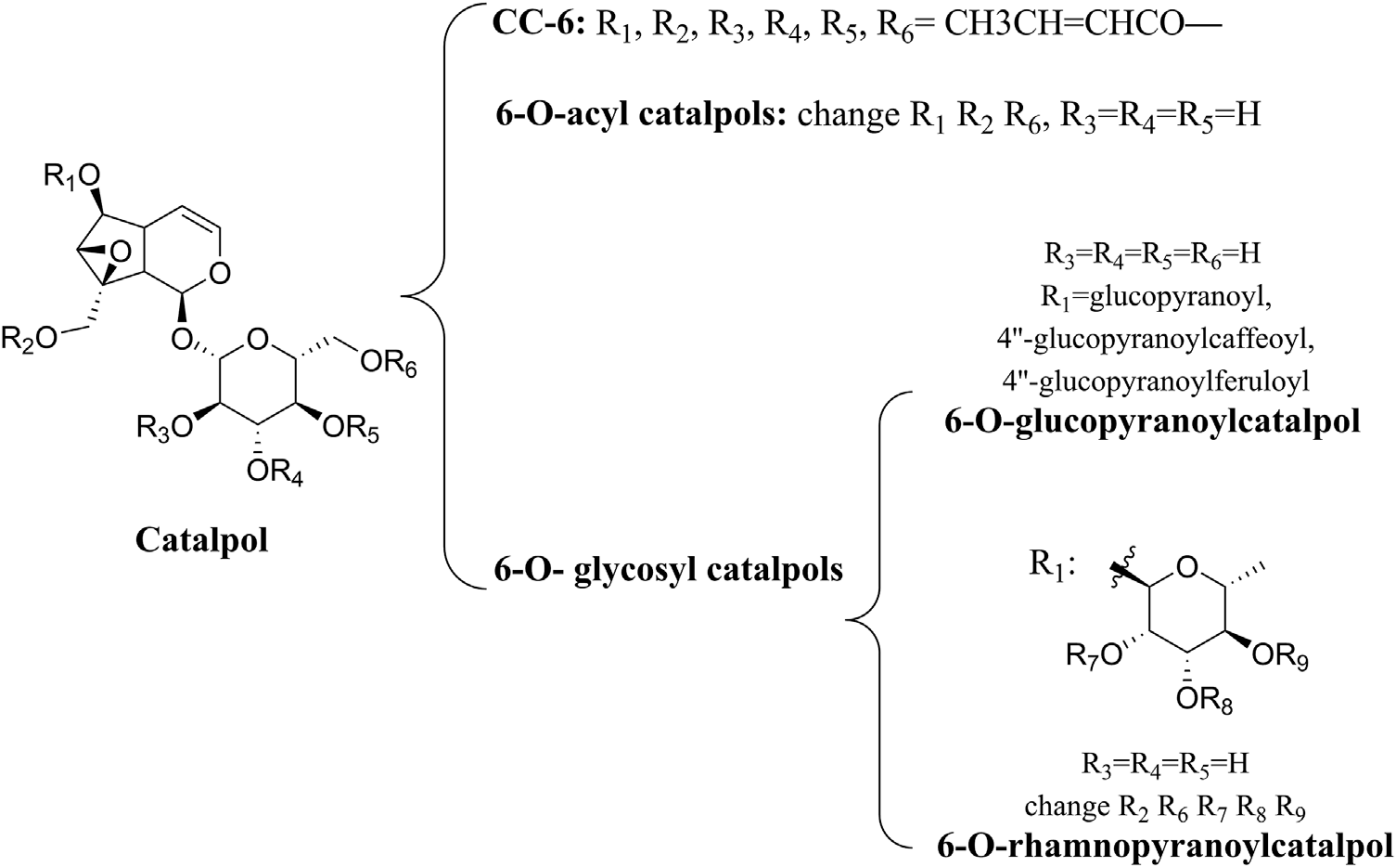
Catalpol and its derivatives.

A number of catalpol derivatives are usually derived from plants. Different substituents at different positions will produce significant functional differences when the main skeletons of compounds are the same. The catalpol derivatives mentioned following have been listed in terms of their chemical structures (Figure 2). In another study, researchers summarized the natural derivatives of catalpol discovered between 1888 and 2018, categorizing them into three categories: 6-O-acyl catalpols, 6-O-glycosyl catalpols, and other derivatives (Zhang et al., 2019a). A wide range of pharmacological activities was observed in these derivatives, but only the picroside mentioned was neuroprotective. The PI3K/Akt pathway was only mentioned by these researchers in the section of cardiovascular protection. As small-molecule drugs, catalpol and its derivatives have obvious disadvantages, such as their hydrophilicity and rapid metabolism, which inhibit their penetration of the blood–brain barrier (Ginter et al., 2014). Therefore, chemical modification of its derivatives must result in improved bioavailability. Liu et al. (2020) used computer-aided drug design (CADD) to dock catalpol crotonates with glutathione peroxidase (GSH-Px) which has neuroprotective properties. By using microwave-assisted synthesis (MWAS) as a synthesis method, they were successful in designing and synthesizing catalpol hexacrotonate (CC-6). The kinetic simulation study of CC-6 indicated that it was a successful combination with GSH-Px, and *in vitro* experiments also showed that it was neuroprotective. In order to determine if it is effective in treating diabetic encephalopathy, more studies need to be conducted *in vivo*.

### Geniposide and its derivatives

As a bioactive iridoid glycoside, geniposide has been isolated from a wide range of plant species, including the Rubiaceae family. As early as 1969, Inouye and Saito were the first to isolate the compound from the gardenia fruit (Inouye et al., 1969). There has been research showing that geniposide activated Akt and then increased PI3K and GSK-3β expression, thus improving pathological symptoms in epileptic rats (Wei et al., 2018). Moreover, Wang et al. (2021b) have confirmed that geniposide could inhibit hippocampal neurons’ apoptosis *via* the aforementioned pathway, resulting in antidepressant properties in the brain. It is well known that neuropathic pain (NP) results from a lesion or disease of the somatosensory system, including peripheral fibers and central nerves (Colloca et al., 2017). As a neurodegenerative disorder, NP is thought to arise from degeneration of inhibitory interneurons in the dorsal horn of the spinal cord, following peripheral nerve injury (Apkarian and Scholz, 2006). Geniposide could inhibit the EGFR/PI3K/Akt signaling pathway, thereby alleviating the pain symptoms in sciatic nerve injury in the chronic constriction injury (CCI) model of NP, which provided a basis for further clinical trials to treat NP (Zhang et al., 2022).

**FIGURE 3:**
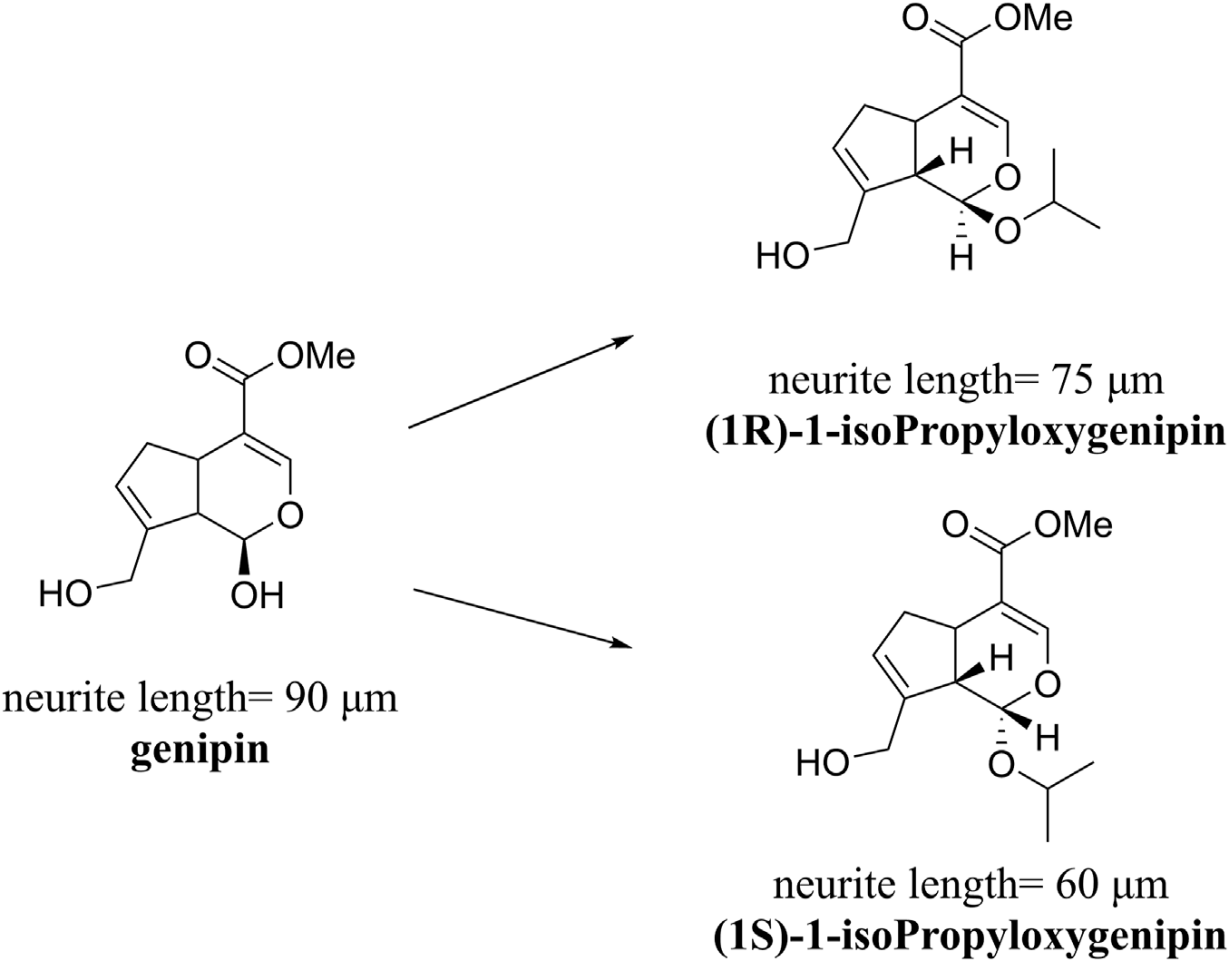
Neurite lengths in PC12 cells were measured after 2 days of treatment with compounds (20 mM). Relative active: genipin ≈ 1-isopropyloxygenipin (Suzuki et al., 2010).

Approximately 90 species of geniposide derivatives exist in nature with various pharmacological properties, including neuroprotective properties. Geniposide can be hydrolyzed into genipin by β-glucosidase *in vivo*. The primary metabolite of genipin and geniposide is genipin sulfate, which is highly toxic in the body (Hou et al., 2008). During the early years, a series of 1-alkyloxygenipins were designed and synthesized based on CADD studies of genipin’s structure and electronic properties. These researchers changed the hydroxyl group of genipin at position 1 into an alkoxy group, thereby obtaining (1R)-isopropyloxygenipin, which had better stability and was physiologically similar to genipin. By converting the hydroxyl group of genipin to the alkoxy group at position 1, they obtained (1R)-isopropyloxygenipin, which exhibits similar physiological properties and has enhanced physiological stability compared to genipin (Figure 3) (Suzuki et al., 2010). Cai et al. (2020) synthesized and designed an acetylated derivative of geniposide, penta-acetyl geniposide [(Ac) 5 GP], which improved the lipophilicity and pharmacological properties of geniposide. In chronic unpredictable mild stress (CUMS) depression rats, (Ac) 5 GP might be effective.

### Gardenamide A and its derivatives

**FIGURE 4:**
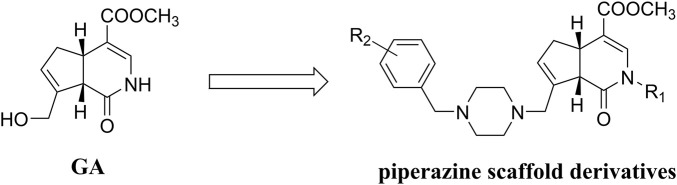
Chemical structure of derivatives for GA based on the piperazine scaffold.

Gardenamide A (GA) is an analog of genipin isolated from *Rothmannia urcelliformis*, which can inhibit cell apoptosis through the PI3K/Akt pathway (Wang et al., 2015b). Novel GA derivatives have been reported in recent years. Some researchers developed this series of derivatives based on the piperazine scaffold (Figure 4), which has shown promise in the development of drugs for neurodegenerative diseases. In the end, four derivative compounds were found to be potent multifunctional neuroprotective agents (Zhang et al., 2019b). We presented a table containing the chemical structures of the four derivatives and the results of their evaluation against each of oxygen–glucose deprivation (OGD)-, hydrogen peroxide (H_2_O_2_)-, and amyloid-β1-42 (Aβ1-42)-induced experimental models and compared with GA (Table 1). The SAR analysis revealed two conclusions: 1) substitution of a 2-isopropyl group on GA resulted in higher cell survival than the substitution of a 2-methyl group in GA; 2) compounds with an electron-withdrawing group on the benzene ring showed better neuroprotective activity.

**TABLE 1:** Chemical structures of the four derivatives and comparison of cell viability of GA with four derivatives at 10 μM (Zhang et al., 2019b).

			Survival rate ± SD (%)		
Comp	R1	R2	OGD	H_2_O_2_	Aβ1–42
1	CH_3_	4-F	85.7 ± 1.0	78.5 ± 0.8	80.8 ± 1.1
2	CH(CH_3_)_2_	H	84.5 ± 1.6	76.2 ± 0.8	76.4 ± 1.0
3	CH(CH_3_)_2_	4-F	86.5 ± 1.1	72.4 ± 0.8	74.0 ± 0.9
4	CH(CH_3_)_2_	4-NO_2_	87.6 ± 1.5	82.1 ± 1.3	84.1 ± 1.7
GA			79.3 ± 1.3	70.9 ± 1.3	71.1 ± 1.3

## Sesquiterpenoids

Among terpenoids, sesquiterpenes are composed of three isoprene units, which can be roughly divided into acyclic and cyclic types according to their chemical structures. The natural products described in the following section are all classified as cyclic sesquiterpenes.

### Bilobalide and its derivatives

The *Ginkgo biloba* (EGb) tree is the oldest species of trees in existence. It has been used as a traditional Chinese medicine for thousands of years and is currently being used worldwide (Nakanishi and chemistry, 2005). The “Encyclopedia of Chinese Herbal Medicines” recorded that it could be used to treat strokes and myocardial infarctions (Chevallier, 2016). In 1970, highly concentrated and stable substances were first extracted from *Ginkgo biloba* leaves by Dr. Willmar Schwabe Pharmaceuticals (Karlsruhe, Germany), who developed a standard extraction method for *Ginkgo biloba* leaves and determined the chemical structure of this extract (Le Bars, 2003). The standardized extract of *Ginkgo biloba* (EGb) usually contains 6% terpene trilactones (TTLs), and bilobalide (BB) is the only sesquiterpene trilactone found in TTLs (van Beek and Montoro, 2009). Several studies have suggested that the neuroprotective properties of the *Ginkgo biloba* extract might be attributable to the presence of BB (Defeudis, 2002; Huang et al., 2003; Jiang et al., 2014). By antagonizing the GABA receptor, BB could increase the levels of glutamic acid decarboxylase (GAD) in the brain, reducing the transmission of GABA in the brain and resulting in an anticonvulsant effect (Sasaki et al., 1999). Furthermore, it could inhibit the release of choline caused by activating phospholipase A2, which reduced the death of neurons (Weichel et al., 1999; Huang et al., 2003; Strømgaard and Nakanishi, 2004).

In recent years, a study has revealed that BB’s neuroprotective properties are mediated by the PI3K/Akt pathway. BB has been shown to exert anti-apoptotic effects by activating this pathway involved in neuronal apoptosis in an *in vitro* experiment model (Shi et al., 2010a). In addition, *in vivo* studies verified that BB supports neuronal cell survival in patients suffering from ischemic stroke (Zheng et al., 2018). Therefore, the development of BB as a treatment for neurodegenerative diseases is worth continuing.

**FIGURE 5:**
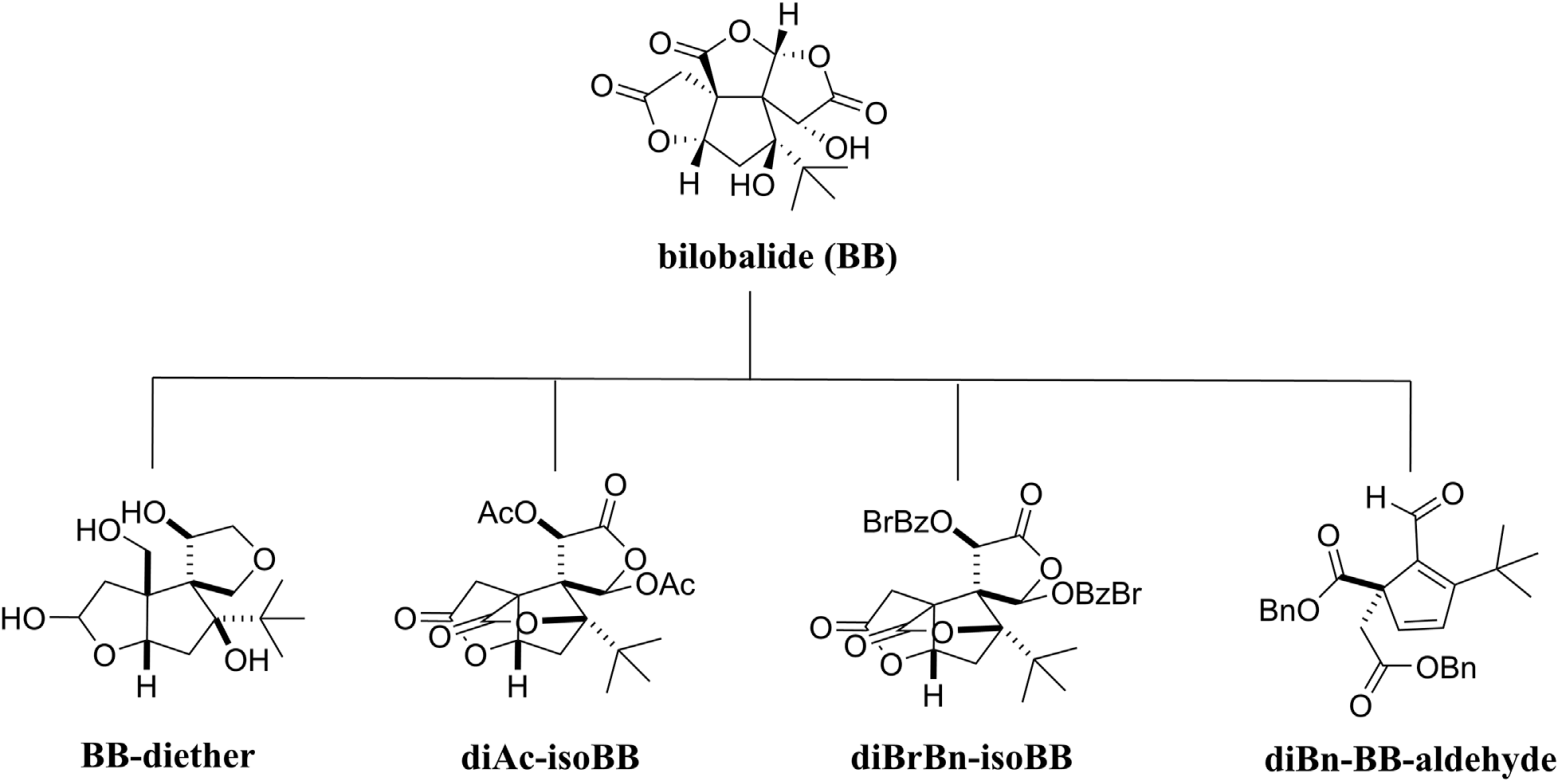
Bilobalide and its derivatives.

Due to the limitations to molecular scaffold reconstruction in medicinal chemistry, BB’s SAR research has not been completed. Recently, some researchers have conducted SAR studies on BB to clarify the relationship between the modification of lactone structure and its biological activity. Four derivatives of BB were synthesized following structural modifications: BB-diether, diAc-isoBB, diBrBn-isoBB, and diBn-BB-aldehyde (Figure 5). *In vitro*, the biological identification found that BB-diether without a lactone group neither protected Aβ (1–40) peptides nor promoted the growth of nerve axons, but other compounds showed positive effects, which proved the importance of lactone groups. Furthermore, both diAc-isoBB and diBrBn-isoBB possessed better activity than BB, indicating that it is beneficial to replace the hydroxyl groups in BB (Usuki et al., 2020).

### Artesunate

Artesunate (ART), a derivative of artemisinin with numerous biological functions, possesses a good permeability to the blood–brain barrier and is usually given intravenously to patients in its water-soluble ionic form (Leicach and Chludil, 2014). A study has shown that ART effectively ameliorated the insufficient endogenous neural stem/progenitor cell (NSPC) proliferation caused by ischemia. By stimulating the PI3K/Akt signaling pathway, ART could increase the phosphorylation level of FOXO-3a, downregulate p27kip1, and inhibit the transcription of FOXO-3a, thus recovering brain function (Zhang et al., 2020). According to a SAR study, the endoperoxide bridge was essential for the activity of ART, but the lactone group was not, and reducing lactone carbonyl to dihydroartemisinin (DART) could improve its solubility (Schmidt, 2006).

### Methyl lucidone

Methyl lucidone (MLC) is extracted from the fruit of *Lindera erythrocarpa* Makino (Lauraceae) distributed in Korea, Taiwan, Japan, and China (Ichino et al., 1988). By activating the PI3K/Akt pathway, MLC could promote phosphorylation of Nrf2 and HO-1 transcription and then play an antioxidant role, which might have therapeutic potential for neurological disorders (Park et al., 2019).

## Diterpenoids

A diterpene consists of four isoprene units and contains 20 carbon atoms. The following is a description of the diterpene ginkgolides.

### Ginkgolides and their derivatives

**FIGURE 6:**
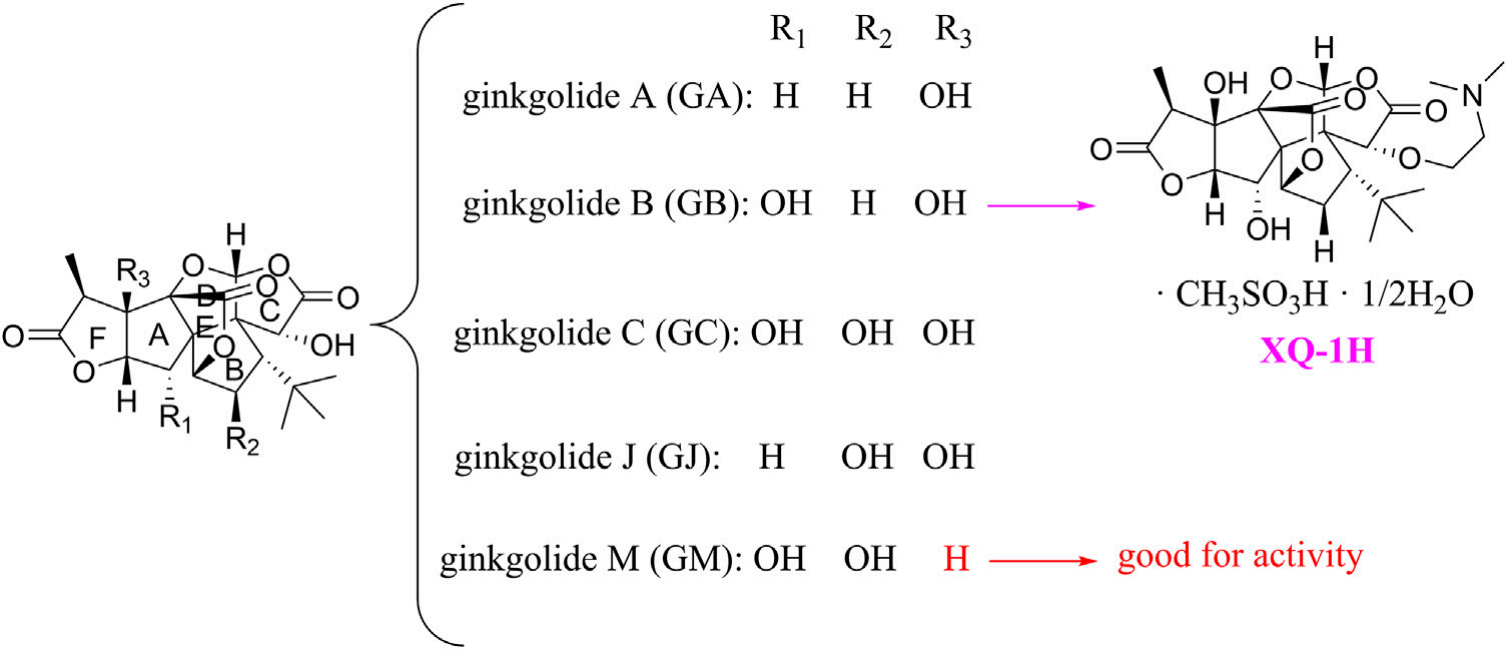
Ginkgolides and their derivatives.

In general, diterpene ginkgolides are known as ginkgolides A, B, C, J, and M, which are rare natural compounds with t-Bu groups. Ginkgolides are also bioactive components of the EGb extract, just like bilobalide. The five main components of ginkgolides differ only in the number and position of their hydroxy groups, and all have six five-membered rings: a spiro [4.4]-nonane carbocyclic ring, three lactones, and a tetrahydrofuran ring (Figure 6). There is no doubt that they are special due to their tert-butyl structure (Strømgaard and Nakanishi, 2004).

The diterpene ginkgolide meglumine injection (DGMI) is often used to treat ischemic stroke in the clinic, whose main component is ginkgolide B (GB). Studies have reported that DGMI significantly activated the PI3K/Akt signaling pathway *in vitro* and *in vivo* to promote cell survival and inhibit apoptosis, which might be one mechanism for its efficacy in stroke treatment (Jin et al., 2017; Zhang et al., 2018; Liu et al., 2019). According to a study *in vitro*, ginkgolide K (GK) had a superior therapeutic potential than GB since GK was easier to upregulate PI3K and p-Akt expression, affecting downstream pathways, thereby contributing to anti-inflammatory and antioxidant effects (Yu et al., 2018b). Furthermore, GK can activate diverse signaling pathways in order to serve a neuroprotective function. For example, some researchers suspected that GK activated the PI3K/Akt pathway to treat PD by upregulating BDNF, but this hypothesis remains unsubstantiated (Yu et al., 2018a).

The results of an early SAR study on GB indicated that it could exert anti-inflammatory and anticoagulant effects by antagonizing the platelet-activating factor receptor (PAFR) (Strømgaard and Nakanishi, 2004). In addition to inhibiting the PAFR, ginkgolides have also been proven to be potent and selective inhibitors of the inhibitory glycine receptor (GlyR), which is a critical target for brain function (Kondratskaya and Krishtal, 2002; Jaracz et al., 2004a). Some researchers selectively derivatized the hydroxyl groups of ginkgolide C (GC) to produce various ether, ester, and carbamoyl derivatives (Jaracz et al., 2004b). The derivatives with only one benzyl substituent at 10-OH had better activity among them, but even the best compound 12 was far less potent than the parent compound GC, so the hydroxyl group was very important to the inhibitory activity of GlyR.

In addition, a study reported the SAR between ginkgolides and different subtypes of GlyR. Due to the previous discovery that lactone structures C and F inhibited GlyR, researchers modified these structures chemically and synthesized four series of derivatives: 1) both lactones were complete and derived from different positions; 2) lactone C had been opened or removed or modified but not the remaining lactones; 3) different lactones had been reduced in the chemical structure; and 4) to derive or oxidize 7-positions. They studied the biological functions of these derivatives *in vitro* and found only six derivatives were effective, confirming that both lactones were essential for inhibiting GlyR. Meanwhile, ginkgolide M (GM) was the most active of all ginkgolide lactones for inhibiting GlyR in a fluorescence-based membrane potential assay. The structural peculiarity of GM was the lack of 3-OH, suggesting that 3-OH was not favorable for GlyR activity. Following this, a 3D pharmacophore model was generated for use in virtual screening. All hit compounds failed to exhibit any activity in pharmacological testing due to their less rigid structures than ginkgolides, indicating that rigid structures were necessary for blocking GlyR (Jensen et al., 2007). 10-O-(N,N-dimethylaminoethyl)-ginkgolide B methane-sulfonate (XQ-1H) (Figure 6) is a derivative of GB (Deng et al., 2009). An *in vivo* study has found that XQ-1H promoted angiogenesis by activating the PI3K/Akt signaling pathway, which is a potential stroke treatment (Fei et al., 2021).

## Triterpenes

Triterpenes are a class of terpenoids constructed from six isoprene units. In general, triterpene saponins are composed of glycosides, sugars, and uronic acids, among which *Panax notoginseng* saponin is a well-studied natural neuroprotectant related to the PI3K/Akt signaling pathway. We mainly discussed asiatic acid and asiaticoside, two triterpenoids that have been extracted from the *Centella asiatica* extract, which are rich in triterpenoids. The pharmacological mechanisms of other reviewed triterpenoids are also related to the PI3K/Akt pathway, but there are only a few medicinal chemistry studies available.

### Alisol A 24-acetate

Alisol A 24-acetate (24A) is a protostane-type tetracyclic triterpenoid compound found in the well-known traditional Chinese medicinal herb *Alisma orientale* (Sam.) Juz. (Chen et al., 2020b). Lu et al. (2021) explored the possible neuroprotective mechanism of 24A, finding that 24A activated the PI3K/Akt signaling pathway to promote the expression of downstream anti-apoptosis proteins, making it an effective treatment for cerebral ischemia-reperfusion injuries. Unfortunately, the chemical properties of 24A are unstable, and it can be converted into 23-acetate by acetylation (Makabel et al., 2008). Currently, there is no new research on improving its structure.

### Polygalasaponin F

Polygalasaponin F (PGSF), an oleanane-type triterpenoid saponin, is the main terpene in *Polygala japonica* Houtt. Zhang et al. (1995a) and Zhang et al. (1995b) isolated it for the first time in 1995 from *Polygala japonica*. It has been reported that PGSF might play an anti-apoptotic role by mediating the PI3K/Akt pathway, and further animal and clinical trials should be conducted to verify the pharmacological effect of PGSF to treat cerebral ischemia (Xie et al., 2020). A study has found that the NR2A-containing NMDAR/PI3K/Akt signaling pathway might be one of the neuroprotective mechanisms by which PGSF inhibits the excitotoxicity of cells in a concentration-dependent manner (Sun et al., 2022).

The development of isolation and structure identification technology has promoted the discovery of active ingredients in *Polygala japonica* Houtt over the past few decades. Although the modification of PGSF has not been reported yet, research studies about separating more triterpene saponins from *Polygala japonica* Houtt have never stopped.

### *Panax notoginseng* saponins

*Panax notoginseng* saponins (PNS) are extracted from the root or rhizome of the herb *Panax notoginseng* (Bruk.) F. H. Chen (Araliaceae). There are five main components of PNS: notoginsenoside R1, ginsenoside Rg1, ginsenoside Re, ginsenoside Rb1, and ginsenoside Rd. PNS can treat vascular and cardiovascular diseases such as ischemic stroke because of its anti-hypoxia, anti-aging, and immune-boosting functions (Yang et al., 2014; Duan et al., 2017). Wu et al. (2020a) demonstrated that the neuroprotective property of PNS was mediated by the EGFR/PI3K/Akt signaling pathway, which was one of the most representative neuroprotective pathways. EGFR was transactivated after an ischemic stroke in an organism and then stimulated downstream signal cascades to protect and promote the growth of cells. An *in vivo* study by Yang et al. (2018) also confirmed that PNS promoted the growth of neurites and plays a neuroprotective role by activating the EGFR/PI3K/Akt pathway. The well-known Xueshuantong for injection (lyophilized) (血栓通注射液, XST) has an excellent curative effect against vascular and cardiovascular diseases, and its main component is PNS.

A study has shown that PNS activated the PI3K/Akt/mTOR pathway in both *in vivo* and *in vitro* experiment models (Pan et al., 2021). By upregulating mTOR, this pathway inhibited autophagy and promoted neuronal survival. In addition, some researchers have studied the therapeutic effect of notoginsenoside R1 (NGR1) on hypoxic-ischemic brain damage (HIBD). They found that NGR1 targeted the endoplasmic reticulum and regulated the PI3K-Akt-mTOR/JNK signaling pathway. As a result of the interaction between the endoplasmic reticulum and PI3K, Akt/mTOR was activated, thus inhibiting JNK downstream of the endoplasmic reticulum. Ultimately, NGR1 could inhibit neuronal apoptosis and promote neuronal survival, which has a significant neuroprotective effect on newborns and contributes to long-term neurological recovery (Tu et al., 2018). Vinaginsenoside R4 (VGN4) is a minor component of *Panax notoginseng* and *Panax japonicus* (Zhu et al., 2016). In an *in vivo* PD model, VGN4 activated the PI3K/Akt/GSK-3β signaling pathway. Then, the NF-κB pathway was triggered to resist an organism’s oxidative stress reaction and control cell apoptosis and proliferation (Luo et al., 2020). Thus, VGN4 has the potential to treat PD and can be further studied later.

**FIGURE 7:**
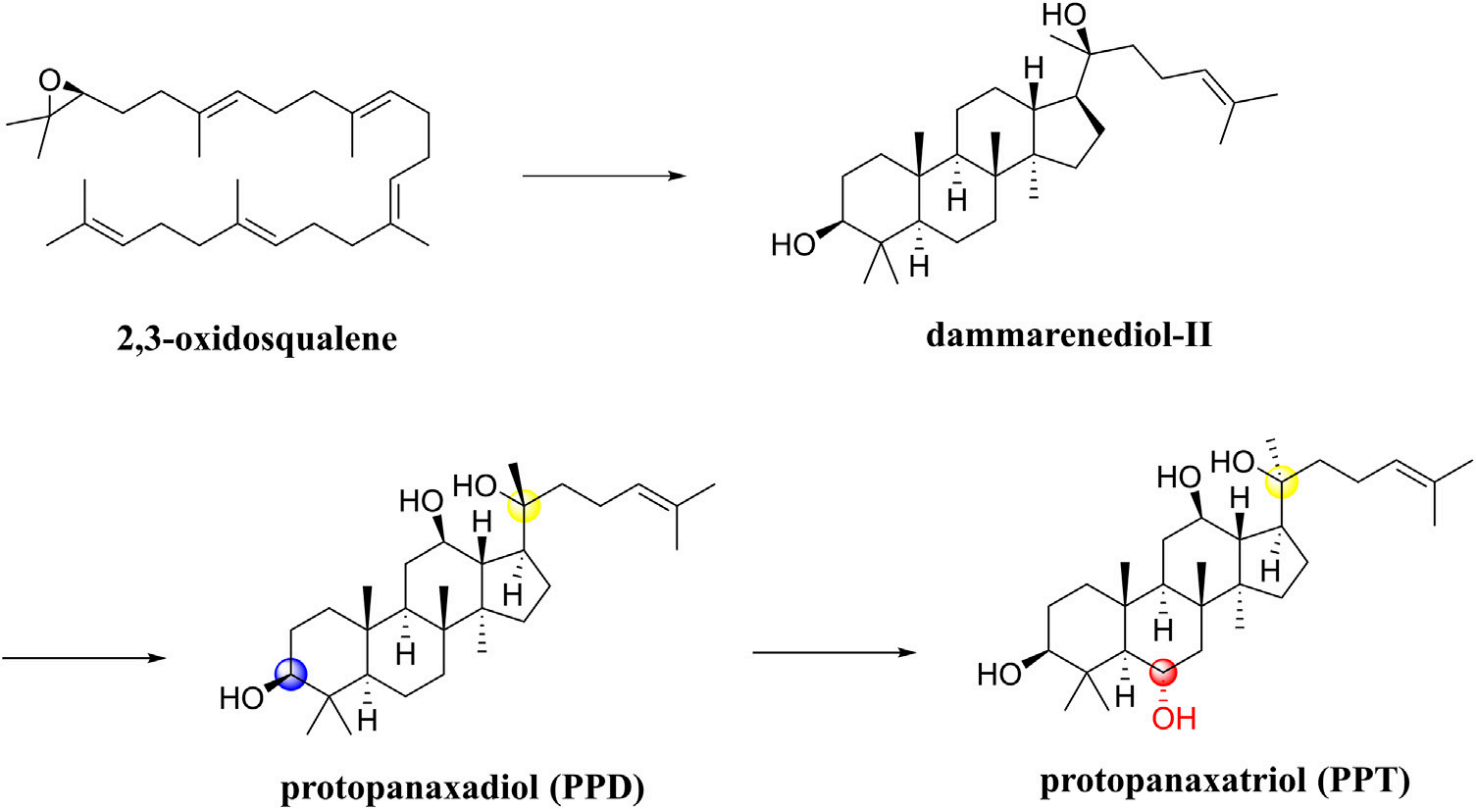
Synthetic routes and different types of notoginseng saponins.

Notoginseng saponins are all dammarane-type saponins, classified as PPDs and PPTs. A first step in the biosynthesis process (Figure 7) involves cyclizing 2,3-oxidosqualene to dammarenediol-II. It is then hydroxylated to produce protopanaxadiol (PPD),β,12β,20-trihydroxydammar-24-ene. On the one hand, O-glycosylation of PPD results in the biosynthesis of various PPD-type saponins, such as ginsenosides Rb1 and Rd. On the other hand, PPD is hydroxylated into protopanaxatriol (PPT) and 3β,6α,12β,20-tetrahydroxydammar-24-ene. Additionally, PPT is biosynthesized through O-glycosylation into PPT-type saponins, such as ginsenoside Rg1 and ginsenoside Re (Shin et al., 2015). In terms of chemical structure, both PPD and PPT types contain hydroxyl groups at C-3, C-12, and C-20 positions, but PPD has one less hydroxyl group at position 6 than PPT. Furthermore, sugar units are linked to them in different positions such that PPD is either at C-3 or C-20, but PPT is either at C-6 or C-20 (Wu et al., 2019b). It is known that *Panax ginseng* processing involves oxidation, hydrolysis, and/or dehydration, which can produce a series of artificial compounds that are biologically active. Some researchers have summarized the structural characteristics of over 100 ginsenosides found in the processing (Shin et al., 2015).

In a SAR study, different saponin chemical backbones have been shown to affect autophagy, which plays a role in neurodegenerative disease pathogenesis. Saponins of the PPT type inhibit autophagy, while saponins of the PPD type and their transformed types promote autophagy (Wu et al., 2019b). Stereoisomers significantly influence PNS’s pharmacological activity in particular. In the processing of raw *Panax notoginseng*, the C-20 position of the saponin is deglycosylated to form optical saponin epimers, and 20(S)-Rg3 is more neuroprotective than 20(R)-Rg3 (Peng et al., 2018).

### Echinocystic acid and its derivatives

Echinocystic acid (EA) is an oleanane pentacyclic triterpenoid with wide pharmacological activity, extracted from plants of *Gleditsia sinensis* Lam. In an *in vivo* study, Chen et al. (2020a) reported that EA served as a neuroprotective agent by activating the PI3K/Akt pathway. It is possible that EA can be used in clinical trials in order to treat intracerebral hemorrhage (ICH).

Due to EA’s complex structure, stable ring system, and limited active sites, few reports have been published regarding its chemical modification. The chemical modification of EA is mainly concentrated in 3-hydroxyl, 16-hydroxyl, C12–C13 double bonds, and 28-carboxyl groups, but their pharmacological effects are anti-inflammation and anti-hepatitis C virus (HCV) (Georgatza et al., 2016; Yu et al., 2016). By coupling EA to cyclodextrin (CD), it is possible to increase EA’s water solubility without causing adverse effects (Jiao et al., 2019). Moreover, Wang et al. (2015a) expanded and opened the rings A and/or C of EA to synthesize a series of derivatives of EA. As a result, this modification could decrease their bioactivity, suggesting that a rigid skeleton is essential for their bioactivity.

In order to improve bioavailability, some researchers wished to obtain related compounds through microbial transformation, followed by activity detection and modifications to the structure. Using the aforementioned method, Feng et al. (2010) have reported that 3-β-OH and 16-α-OH of EA could be distinguished for the first time, providing new insights into the structural modification of EA. As compared to traditional chemical methods, microbial transformations have greater advantages in oxidizing unactivated C–H bonds, and we can activate saturated C–H bonds of EA to expand its structural diversity and develop its pharmaceutical properties (Xu et al., 2017). Some studies have found that a new method of biotransformation could provide EA derivatives with cleavage and 7-member-lactone of ring A and derivatives with single or double hydroxylations, proving that this method could overcome the shortcomings of traditional chemical modification methods (Fu et al., 2018; Fu et al., 2019).

### Asiatic acid and asiaticoside

A plant belonging to the family Apiaceae, *Centella asiatica* (L.) Urban, is inexpensive and easy to obtain as a medicinal remedy. Historically, ayurvedic practitioners have used it as a neuroprotective agent, and its major components are asiatic acid (AA) and asiaticoside (AS). Early evidence has suggested that AS could be used to treat PD and dementia (Xu et al., 2012; Lin et al., 2013; Chen et al., 2014). In an *in vitro* PD model, AS inhibited the decrease in PI3K expression induced by rotenone, eliminating ROT-induced abnormal neuronal dynamics at the synapse, thus affecting PI3K/Akt and thus activating the cytoprotective pathway to treat PD (Gopi et al., 2017). Both *in vivo* and *in vitro* studies have demonstrated that AA activated the PI3K/Akt pathway, which inhibited the activity of GSK-3β and reduced neuronal apoptosis (Nataraj et al., 2017; Cheng et al., 2018). It is known that diabetic encephalopathy is a complication of diabetes that causes cognitive impairment. The molecular mechanism of *Centella asiatica* for treating this disease has been reported in recent years. A study of AS has demonstrated that the compound upregulated the expression of synaptic proteins through the PI3K/Akt/NF-κB pathway *in vitro*, helping to restore synaptic connectivity (Yin et al., 2015). In recent years, some researchers have conducted *in vivo* experiments with an aqueous extract of the *C. asiatica* leaf aqueous extract in the diabetes rat model and found that PI3K and Akt levels increased in the rat brain, prolonging the survival time of cells (Giribabu et al., 2020).

**FIGURE 8:**
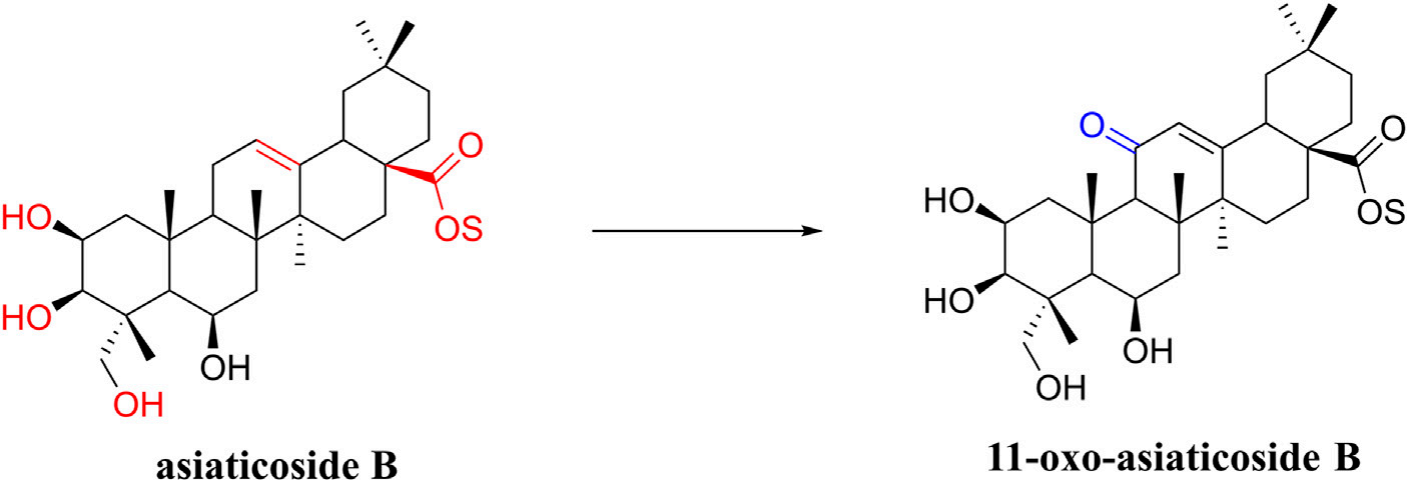
Asiaticoside B and its derivatives.

In a study on the chemical composition of *Centella asiatica* extracts, Wu et al. (2020b) identified six new compounds, three pairs of oleanolic and ursolic pentacyclic triterpenoid isomers. Among these compounds, compound III was similar to asiaticoside B in chemical structure and was named 11-oxo-asiaticoside B (Figure 8). Compound III inhibited oxidative stress response, reduced apoptosis, and played a neuroprotective role through the PI3K signaling pathway. In the future, compound III should be further developed in order to explore its therapeutic potential.

So far, many derivatives have been synthesized by chemical modification of the structure of asiaticoside, usually on C-2, C-3, and C-23 hydroxyl groups, a C-12 olefin group, and a C-28 carboxylic acid group, which generally have less toxicity, better bioavailability, and efficacy (Lv et al., 2018; Nagoor Meeran et al., 2018). These derivatives can treat cancer, diabetes, and other diseases. In the future, we will also need to work on developing such derivatives for neurological disorders.

### Pachymic acid

Pachymic acid (PA) is a natural triterpene of *Poria cocos* origin and a derivative of the lanostane skeleton (Zheng and Yang, 2008; Ríos, 2011). PA inhibited neuronal apoptosis by downregulating the expression of caspase-3, following activation of the PI3K/Akt pathway to reduce brain damage (Pang et al., 2020). In the future, PA may be used to treat hemic cerebrovascular diseases at an early stage. Furthermore, Zhu et al. (2021) have described the PA’s synthetic pathway and the genes involved in the synthesis. PA and ganoderic acid are homologous derivatives with highly similar carbon skeletons, so they looked for inspiration in the synthesis of *G. lucidum* and discovered that *WcSOAT* gene plays a role in the biosynthesis of PA in *Wolfiporia cocos*.

### Stellettin B

A marine organism-derived compound, stellettin B (SB), is an isomalabaricane triterpenoid found in the sponge *Jaspis stellifera*. SB has been shown to reverse the downregulation of the PI3K/Akt signaling pathway caused by 6-OHDA *in vitro* and to reverse ataxia in a zebrafish model of PD *in vivo* (Feng et al., 2019). Therefore, SB is expected to become a leading anti-apoptotic and antioxidant compound for the treatment of PD. Interestingly, a study using a homogenous time-resolved fluorescence (HTRF) assay suggested that SB might target a signal protein upstream of the Akt pathway rather than PI3K (Wu et al., 2019a). The SAR analysis of SB revealed that the lactone rings with α,β-unsaturated carbonyl groups were essential for its efficacy (Song et al., 2018).

## Tetraterpenes

A tetraterpene is a class of terpenoids composed of eight isoprene units. In general, tetraterpenoids refer to carotenoids.

The tetraterpene pigments carotenoids, including lycopene and astaxanthin, are found in photosynthetic bacteria, some species of archaea and fungi, algae, plants, and animals. Over the past decade, a number of studies have demonstrated the potential to act as neuroprotective agents for carotenoids.

### Lycopene

Lycopene is a carotenoid of wide biological activity, which is mainly found in tomatoes and other red fruits, and has a stronger antioxidant effect than beta-carotene. There is no doubt that its neuroprotective properties are well known. Huang et al. (2019) found that lycopene reversed the neurotoxicity induced by tert-butyl hydroperoxide (t-BHP) in an *in vitro* AD model and elucidated that its neuroprotective mechanism involved the PI3K/Akt pathway. Recently, researchers have developed a rapid and efficient HPLC-DAD-MS2 method to isolate and characterize some carotenoids and their geometrical isomers from tomato products, including some derivatives of lycopene first discovered such as di-hydroxy cyclolycopene adduct and di-methoxy lycopene (Daood et al., 2022).

### Astaxanthin

Astaxanthin (AST) is a marine carotenoid commonly found in crustaceans (shrimp and crab) (Zarneshan et al., 2020). Both *in vitro* and *in vivo* studies have demonstrated that AST was neuroprotective. AST exerts anti-apoptotic and neuroprotective effects by upregulating the phosphorylation of PI3K and Akt, which can treat neurological diseases such as PD, AD, and ischemic stroke (Wang et al., 2018; Deng et al., 2019). Adonixanthin and adonirubin are intermediates in the synthesis of AST, and like AST, they can improve brain damage associated with ICH by inhibiting lipid peroxidation and quenching singlet oxygen. The antioxidant and anti-inflammatory effects of adonixanthin are attributed to its characteristic structure, in which polyene (conjugated double bond) is the key to turning singlet oxygen into stable triplet oxygen. Additionally, there are differences in the ionone rings between adonixanthin and adonirubin compared to AST, resulting in structural polarity, which contributes to a stronger neuroprotective effect (Maoka et al., 2013; Kishimoto et al., 2016; Iwata et al., 2018).

## Discussion and prospects

So far, in many studies, terpenoids have been found to exert neuroprotective effects by modulating the PI3K/Akt pathway (Table 2), of which triterpenoids account for the majority.

**TABLE 2:** Experimental evidence supporting the neuroprotective role of terpenoids in various neurological disorders.

Scientific name	Chemical formula	Disease	Study type	Does and route	Experimental model	Animal/cell lines	Ref
Monoterpenes							
Catalpol	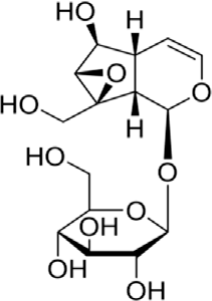	Depression	*in vivo*	5,10, and 20 mg/kg; i.g; 14 days	Streptozotocin-induced hyperglycemic mice	Kunming mice (18–22 g, adult male, 3–4 weeks old)	Wu et al. (2021)
		Stroke	*in vitro* and *in vivo*	2.5, 5, and 7.5 mg/kg; i.p; 7 days	Stroke model or the primary neurons from the rat stroke model	Sprague–Dawley (220–250 g, male and female) rats at youth stage or Sprague–Dawley rats (female and male rats, 200–250 g)	Wang et al. (2019) and Zhu et al. (2019)
Geniposide	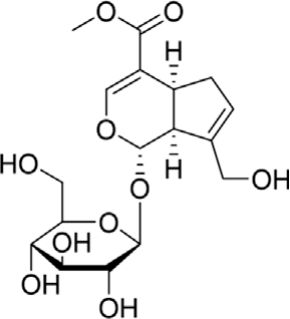	Epilepsy	*in vivo*	0, 5, 10, or 20 mg/kg; i.g., 4 weeks	Maximal electric shock-induced mouse model	C57Bl/6 mice aged 11–12 weeks (20–23 g, male)	Wei et al. (2018)
		Depression	*in vitro* and *in vivo*	0, 30, 60, and 90 mg/kg, i.g., 4 weeks; 50 μM	Chronic unpredictable mild stress (CUMS)-induced depression mouse model; C16 ceramide-induced apoptosis of the hippocampal neuron model	ICR mice (18–22 g, male) and primary hippocampal neurons	
		Neuropathic pain	*in vivo*	0 or 10 mg/kg; i.p; six times a week for 4 weeks	CCI model	Sprague–Dawley rats (250–300 g, male)	Zhang et al. (2022)
Sesquiterpenoids							
Bilobalide	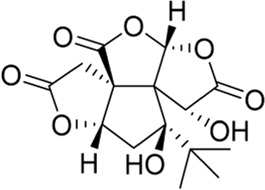	Stroke	*in vivo*	5, 10, or 20 mg/kg; intraduodenal administration	Rat model of middle cerebral artery occlusion (MCAO)	Sprague–Dawley rats (220–230 g; male)	Zheng et al. (2018)
		Neurological disorders	*in vitro*	0, 5, and 10 μM	External factor-induced cell apoptosis model	SH-SY5Y cell line from brains of 0–1-day-old postnatal Sprague–Dawley rats	Shi et al. (2010a)
Artesunate	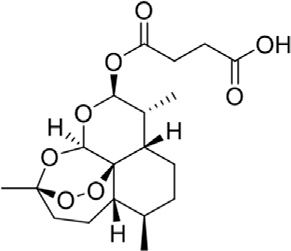	Stroke	*in vitro* and *in vivo*	0, 50, 150, and 250 mg/kg, i.p., 14 days; 0, 0.4, 0.8, and 1.6 μmol/L; 72 h	Rat model of middle cerebral artery occlusion (MCAO) and OGD/R cell model	C57BL/6 mice (newborn) and primaryneural stem/progenitor cells (NSPCs)	Zhang et al. (2020)
Methyl lucidone	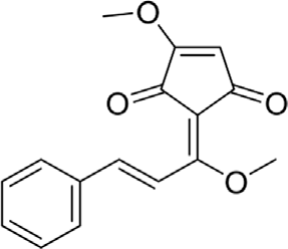	Neurological disorders	*in vitro*	0.5, 1, 3, and 5 µM	Glutamate-induced oxidative stress model	HT-22 cell line	Park et al. (2019)
Diterpenoids							
Ginkgolide B	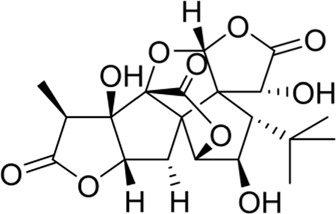	Stroke	*in vitro* and *in vivo*	1, 2, and 4 mg/kg, i.p., twice a day; 0.39-50 mg/L, 0.5-8 h	Rat model of middle cerebral artery occlusion (MCAO) and OGD/R cell model	Sprague–Dawley rats (280–320 g; male) and SH-SY5Y cells	Liu et al. (2019)
Ginkgolide K	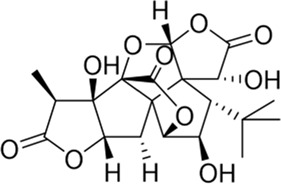	Neurological disorders	*in vitro*	2, 10, 30, and 50 ug/ml	OGD/R cell model	primary cortical astrocytes from newborn mice	Yu et al. (2018b)
Triterpenes							
Alisol A 24-acetate	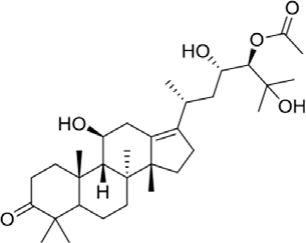	Stroke	*in vivo*	30 mg/kg, i.g, 7 days, i.p., the first 3 days	Global cerebral ischemia (GCI) model	C57BL/6J mice (25–30 g, male, 12 weeks)	Lu et al. (2021)
Polygalasaponin F	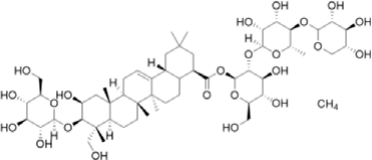	Cerebral ischemia	*in vitro*	0.1, 1, and 10 μmol/L	OGD/Rcell model	Primary cortical neurons from Sprague–Dawley male rats (250–300 g, male) and PC12 cells	Xie et al. (2020)
		Glutamate excitotoxicity	*in vitro*	10 μmol	Glutamate-induced cytotoxicity cell model	Hippocampal neurons from embryonic day 17–19 Wistar rats (200 g, female)	Sun et al. (2022)
Notoginsenoside R1	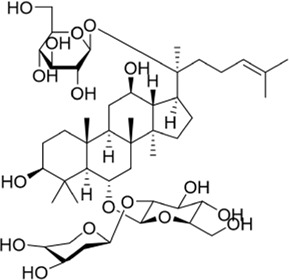	Hypoxic-ischemic brain damage (HIBD)	*in vitro* and *in vivo*	15 mg/kg, i.p, q 12 h, 2 days; 10 μmol/L	Hypoxic-ischemic brain damage model; OGD/Rcell model	Sprague–Dawley rats (7-day-old, male) and rat fetuses (18 days)	Tu et al. (2018)
Vinaginsenoside R4	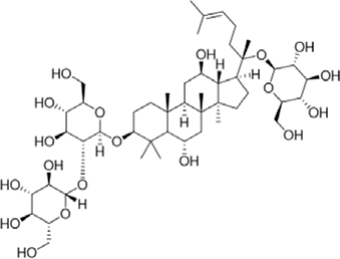	PD	*in vitro*	25, 50, or 100 μM	6-OHDA-induced PD cell model	PC12 cells	Luo et al. (2020)
Echinocystic acid	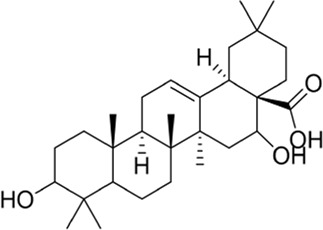	Intracerebral hemorrhage (ICH)	*in vivo*	50 mg/kg, i.p. q.d, 3 days	Collagenase-induced ICH mouse model	ICR mice (25–30 g, male, 8–10 weeks)	Chen et al. (2020a)
Asiatic acid	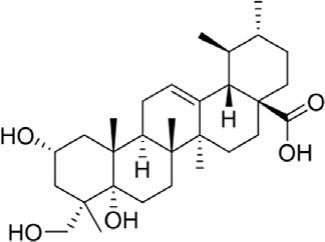	PD	*in vivo*	25, 50, and 100 mg/kg, b.w, 35 days	MPTP/probenecid mouse model of PD	C57BL/6 mice (25–30 g, male)	Nataraj et al. (2017)
		AD	*in vitro*	5, 10, or 20 μM	Aβ25–35-induced neurotoxicity cell model	PC12 cell	Cheng et al. (2018)
Asiaticoside	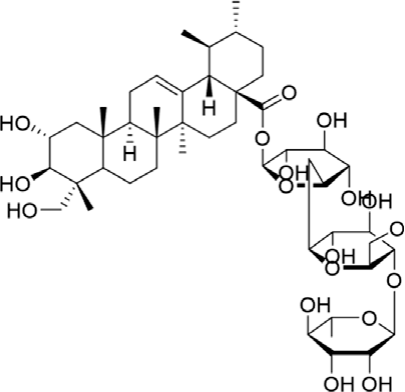	PD	*in vivo*	1 ml/kg, p.o, 0–7 days; 50 mg/kg, p.o, 8–21 days	Rotenone-induced PD rat model	Sprague–Dawley rats (250–300 g, male)	Gopi et al. (2017)
		Diabetic encephalopathy	*in vitro* and *in vivo*	20 or 40 mg/kg, p.o, 30 days; 0.1, 1 μmol/L	Streptozotocin (STZ)-induced diabetic cognitive-deficit rat model; cell model prolonged high glucose incubation	Sprague–Dawley rats (160–180 g, male) and human neuroblastoma SH-SY5Y cells	Yin et al. (2015)
		Diabetic encephalopathy	*in vivo*	50, 100, and 200 mg/kg, p.o, 28 days	Streptozotocin (STZ)-induced diabetic cognitive deficit rat model	Wistar rats (180 ± 20 g, male, 12 weeks old)	Giribabu et al. (2020)
Pachymic acid	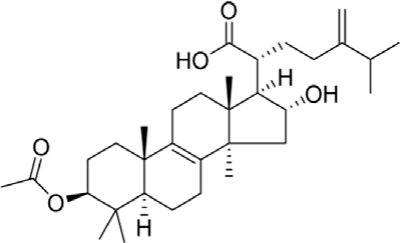	Ischemic cerebrovascular disease	*in vivo*	12.5, 25, 50, and 100 mg/kg, i.p, 24 h	Focal cerebral ischemia/reperfusion (I/R) model	Sprague–Dawley rats (200–220 g, male, 7–8 weeks old)	Pang et al. (2020)
Stellettin B	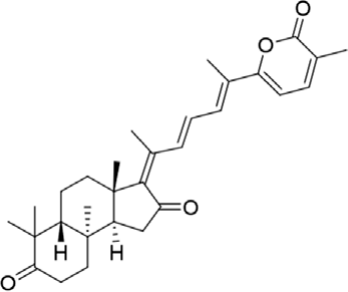	PD	*in vitro* and *in vivo*	1 nM; 0.1, 1, 10, or 100 nM	6-OHDA-induced model of PD	Zebrafish (*Danio rerio*) and SH-SY5Y cells	Feng et al. (2019)
Tetraterpenes							
Lycopene	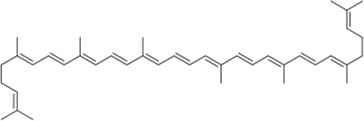	AD	*in vitro*	0, 0.5, 1, 2, 4, and 8 μM	t-BHP-induced neuronal damage model	Primary mouse cerebrocortical neurons from newborn C57BL/6J mice (within 24 h after birth)	Huang et al. (2019)
Astaxanthin	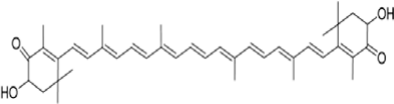	Status epilepticus (SE)	*in vivo*	30 mg/kg, i.p	Pilocarpine-induced status epilepticus rat model	Wistar rats (160–180 g, male)	Deng et al. (2019)
		Neurological disorders	*in vitro*	0.5–10 μM	Hcy-induced neurotoxicity model	Primary rat hippocampal neurons	Wang et al. (2018)

In terms of pharmacology, the neuroprotective effects of some terpenoid natural products have not been fully proven, so it is crucial to establish appropriate experimental models to determine the efficacy of natural products, so those good drug candidates may be identified. In terms of chemical structures, CADD can be used to study chemical structures systematically, such as the studies of catalpol, geniposide, and ginkgolide derivatives previously discussed. The SAR analyses of catalpol and ginkgolides are based on computer simulations of binding to the relevant receptors. To make chemical modifications of ginkgolides, researchers have even established pharmacophore models. CADD can instruct us to make chemical modifications based on the structures of known compounds, improving the properties of compounds, such as enhancing bioavailability, reducing toxicity, and changing solubility. Derivatives of artesunate, methyl lucidone, polygalasaponin F, pachymic acid, and stellettin B are still less studied.

It is sometimes difficult to develop new drugs using traditional chemical modification methods. Researchers may be able to gain some new insights from EA’s use of novel microbial transformations in order to obtain new compounds. In addition, new natural products can be discovered more efficiently by developing advanced separation and characterization technologies.

Thus, we generally have three ideas for developing new neuroprotective terpenoid natural products: 1) exploring the neuroprotective properties of existing natural terpenoids; 2) extracting and isolating new terpenoids from natural herbs that extracted terpenoids with neuroprotective effects; and 3) modifying known terpenoid natural neuroprotective agents chemically to develop small-molecule new terpenoid drugs.

In conclusion, future research should employ comprehensive *in vivo* and *in vitro* experiments to illustrate the key role of PI3K/Akt pathways in neuroprotection for natural products, followed by scientific clinical trials. Developing derivatives of natural products will also promote the discovery of new neuroprotective drugs.
